# Research Collaboration and Outcome Measures of Interventional Clinical Trial Protocols for COVID-19 in China

**DOI:** 10.3389/fpubh.2020.554247

**Published:** 2020-09-02

**Authors:** Ya Gao, Kelu Yang, Ming Liu, Yamin Chen, Shuzhen Shi, Fengwen Yang, Jinhui Tian

**Affiliations:** ^1^Evidence-Based Medicine Center, School of Basic Medical Sciences, Lanzhou University, Lanzhou, China; ^2^Evidence-Based Nursing Center, School of Nursing, Lanzhou University, Lanzhou, China; ^3^Evidence-Based Medicine Center, Tianjin University of Traditional Chinese Medicine, Tianjin, China; ^4^Key Laboratory of Evidence-based Medicine and Knowledge Translation of Gansu Province, Lanzhou University, Lanzhou, China

**Keywords:** COVID-19, SARS-CoV-2, clinical trials, protocol, research collaboration, outcome measures

## Abstract

**Background:** Research collaboration of registered clinical trials for Coronavirus Disease 2019 (COVID-19) remains unclear. This study aimed to analyze research collaboration and distribution of outcome measures in registered interventional clinical trials (ICTs) of COVID-19 conducted in China.

**Methods:** The International Clinical Trials Registry Platform, China Clinical Trials Registry, and Clinicaltrials.gov were searched to obtain COVID-19-registered ICTs up to May 25, 2020. Excel 2016 was used to perform a descriptive statistical analysis of the extracted information. VOSviewer 1.6.14 software was used to generate network maps for provinces and institutions and create density maps for outcomes.

**Results:** A total of 390 ICTs were included, and the number of daily registrations fluctuated greatly. From 29 provinces in China, 430 institutions contributed to the registration of ICTs. The top three productive provinces were Hubei (160/390, 41.03%), Shanghai (60/390, 15.38%), and Beijing (59/390, 15.13%). The top three productive institutions were Tongji Hospital, Tongji Medical College, Huazhong University of Science and Technology (30/390, 7.69%), Zhongnan Hospital of Wuhan University (18/390, 4.62%), and Wuhan Jinyintan Hospital (18/390, 4.62%). Collaborations between provinces and institutions were not close enough. There were many interventions, but many trials did not provide specific drugs and their dosage and treatment duration. The most frequently used primary outcome was Chest/lung CT (53/390, 13.59%), and the most frequently used secondary outcome was hospital stay (33/390, 8.46%). There was a large difference in the number of outcomes, the expression of some outcomes was not standardized, the measurement time and tools for some outcomes were not clear, and there was a lack of special outcomes for trials of traditional Chinese medicine.

**Conclusions:** Although there were some collaborations between provinces and institutions of the current COVID-19 ICT protocols in China, cooperation between regions should be further strengthened. The identified deficiencies in interventions and outcome measures should be given more attention by future researchers of COVID-19.

## Introduction

The severe acute respiratory syndrome coronavirus 2 (SARS-CoV-2), a novel enveloped RNA betacoronavirus, has the characteristics of fast spread and strong infectivity (1–3). In late December 2019, the Coronavirus Disease 2019 (COVID-19) caused by SARS-CoV-2 first appeared, and it then quickly spread to various countries (4–6). On March 11, 2020, the World Health Organization (WHO) declared the outbreak of SARS-CoV-2 as a pandemic (7). As of July 12, 2020, a total of 12,552,765 confirmed cases were reported worldwide, including 561,617 deaths (8). To find an effective drug to treat COVID-19, medical workers and scientific researchers actively carry out research and have registered numerous clinical trials. Recently, scholars have assessed the characteristics and status quo of registered COVID-19 clinical trials (9, 10). However, no research has focused on the research collaboration of these registered clinical trials. This study was designed to evaluate the cooperation between institutions and the distribution of outcome measures in registered interventional clinical trials (ICTs) of COVID-19 conducted in China, to provide a reference for future researchers to register and carry out COVID-19 clinical trials.

## Materials and Methods

### Data Sources

We systematically searched the International Clinical Trials Registry Platform (ICTRP, https://www.who.int/ictrp/en/), China Clinical Trials Registry (ChiCTR, http://www.chictr.org.cn), and ClinicalTrials.gov to obtain registered trials related to COVID-19. The searches were conducted initially on February 20, 2020 and updated on May 25, 2020. The search terms included severe acute respiratory syndrome coronavirus 2, SARS-CoV-2, new coronavirus, new coronary pneumonia, NCP, 2019-nCoV, COVID-19, novel corona virus, novel coronavirus, nCoV-2019, corona virus pneumonia disease 2019, novel coronavirus pneumonia, 2019 novel coronavirus, coronavirus disease 2019, and coronavirus disease-19.

### Inclusion and Exclusion Criteria

We included registered ICTs of COVID-19 that conducted in China without restricting the types of interventions, comparisons, and outcomes. We excluded trials conducted outside China. Studies of basic science, diagnostic test, and epidemiological research as well as duplication and retracted records were also excluded.

### Study Selection and Data Extraction

Two researchers (Y.G. and K.L.Y.) independently reviewed the records and screened out eligible ICTs according to the inclusion and exclusion criteria, and then proceeded to a cross-check. Conflicts were settled through discussions with a third reviewer (J.H.T.). We developed a data extraction form using Microsoft Excel 2016 (Microsoft Corp, Redmond, WA, www.microsoft.com) through discussions with the review team. Then, one author (Y.G., K.L.Y., or M.L.) extracted data from the included ICTs using the pre-defined form and a second reviewer (F.W.Y, or J.H.T.) checked the extracted data. The detailed data included: registration number, registration time, title, inclusion criteria, exclusion criteria, gender and age of the population, sample size, provinces, institutions, interventions, primary outcomes, and secondary outcomes.

### Data Management and Analysis

For institutions, interventions, and outcomes with different expressions, we have processed them, leaving only a standardized name. Microsoft Excel 2016 (Microsoft Corp, Redmond, WA, www.microsoft.com) was used to perform descriptive statistical analysis of the extracted information. VOSviewer 1.6.14 (Leiden University, Leiden, Netherlands) software was utilized to extract provinces and institutions and generate corresponding cooperation network maps. Furthermore, we created density maps for high-frequency primary and secondary outcome measures. In this study, the nodes in the network map represented the analyzed elements (provinces and institutions), the size of the nodes reflected the frequency of elements, the colors of nodes and lines represented different clusters, and the links between nodes indicated the relationship of cooperation or co-occurrence (11–14). The parameters of the VOSviewer were as follows: counting method (fractional counting), ignore documents with many authors (maximum number of authors per document is 25).

## Results

### Screening Results

A total of 3,541 records were retrieved through the systematic literature search, and 1,159 were non-interventional trials. After reading the detailed registration information, we further excluded 1,992 records for the following reasons: trials conducted outside China (*n* = 1,336), duplicate records (*n* = 609), retracted/terminated trials (*n* = 47). Finally, 390 ICTs were included for analysis. The flowchart of the screening process is provided in Figure S1.

### General Characteristics of Included ICTs

**Figure 1:**
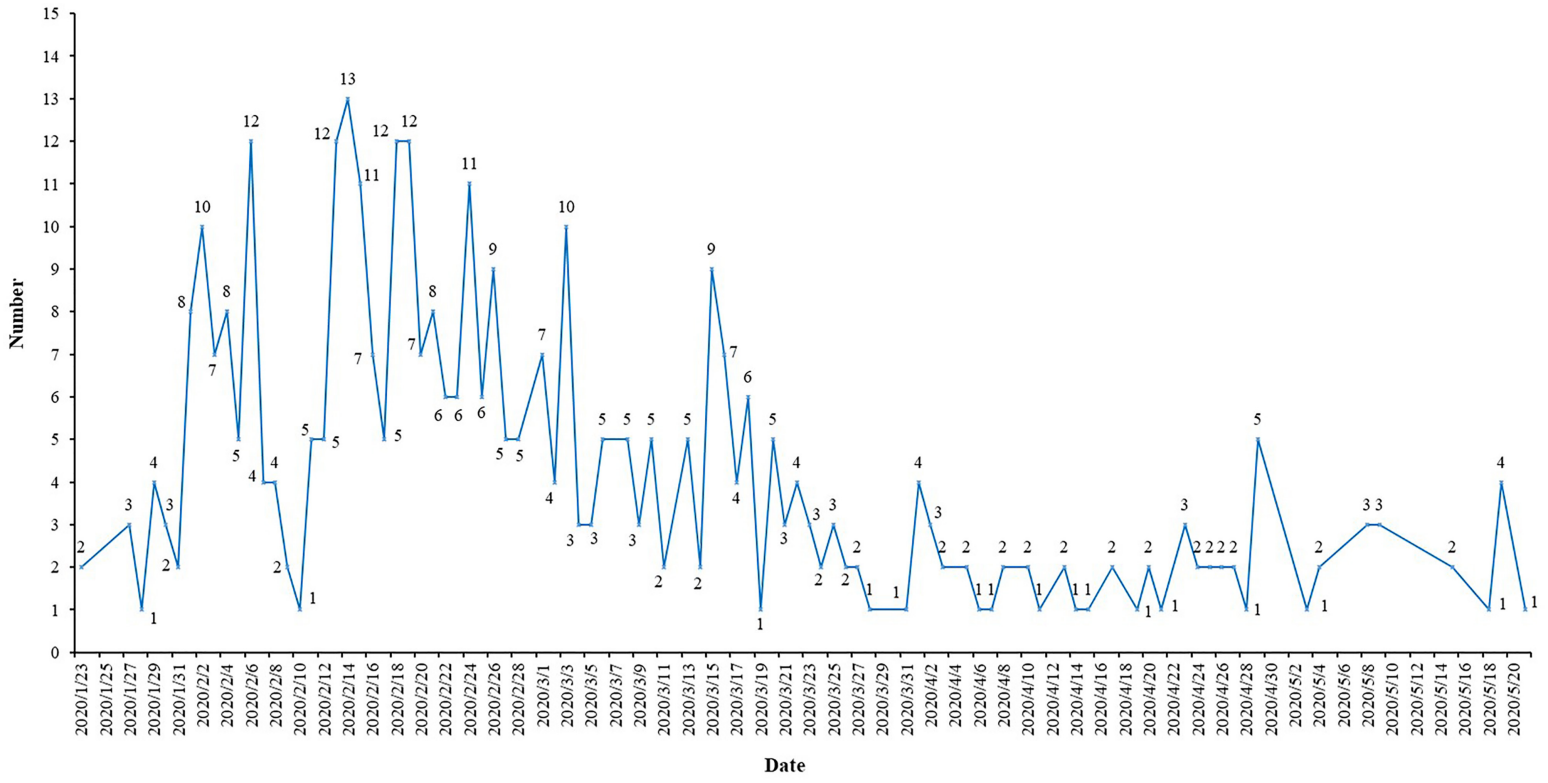
Registration time for ICTs of COVID-19.

The number of daily COVID-19 ICT registrations fluctuated considerably, and the maximum number of registrations per day was 13 (Figure 1). Six (1.54%) ICTs incorporated only males, and the remaining 384 (98.46%) ICTs included both males and females. A total of 74.87% of ICTs included adults (18 years and older), but 59 (15.13%) ICTs did not report the age of the included population. The total sample size of the 390 ICTs was 109,372, and the smallest sample size was only four; the maximum was 20,000, and the median was 100.

### Provinces

A total of 29 provinces participated in the registration of COVID-19 ICTs. The number of ICTs conducted by one, two, three, four, five, and six provinces were 304/390 (77.95%), 61/390 (15.64%), 12/390 (3.08%), 4/390 (1.03%), 6/390 (1.54%), and 3/390 (0.77%), respectively. The top five productive provinces were Hubei (160/390, 41.03%), Shanghai (60/390, 15.38%), Beijing (59/390, 15.13%), Guangdong (44/390, 11.28%), and Zhejiang (34/390, 8.72%); the provinces participating in the registration of six to 21 ICTs were Sichuan (21/390, 5.38%), Jiangsu (18/390, 4.62%), Henan (17/390, 4.36%), Anhui (13/390, 3.33%), Hunan (13/390, 3.33%), Jiangxi (13/390, 3.33%), Heilongjiang (11/390, 2.82%), Shaanxi (11/390, 2.82%), Shandong (8/390, 2.05%), Chongqing (7/390, 1.79%), Fujian (6/390, 1.54%), and Liaoning (6/390, 1.54%). The remaining provinces participated in the registration of fewer than six ICTs, the detailed information is presented in Table 1.

**Table 1:** Provinces contributed to the registration of COVID-19 ICTs [N (%)].

**Rank**	**Provinces**	**N (%)**	**Rank**	**Provinces**	**N (%)**
1	Hubei	160 (41.03%)	16	Fujian	6 (1.54%)
2	Shanghai	60 (15.38%)	17	Liaoning	6 (1.54%)
3	Beijing	59 (15.13%)	18	Guizhou	5 (1.28%)
4	Guangdong	44 (11.28%)	19	Tianjin	4 (1.03%)
5	Zhejiang	34 (8.72%)	20	Hebei	3 (0.77%)
6	Sichuan	21 (5.38%)	21	Guangxi	2 (0.51%)
7	Jiangsu	18 (4.62%)	22	Inner Mongolia	2 (0.51%)
8	Henan	17 (4.36%)	23	Ningxia	2 (0.51%)
9	Anhui	13 (3.33%)	24	Shanxi	2 (0.51%)
10	Hunan	13 (3.33%)	25	Hainan	1 (0.26%)
11	Jiangxi	13 (3.33%)	26	Hong Kong	1 (0.26%)
12	Heilongjiang	11 (2.82%)	27	Jilin	1 (0.26%)
13	Shaanxi	11 (2.82%)	28	Xinjiang	1 (0.26%)
14	Shandong	8 (2.05%)	29	Yunnan	1 (0.26%)
15	Chongqing	7 (1.79%)			

**Figure 2:**
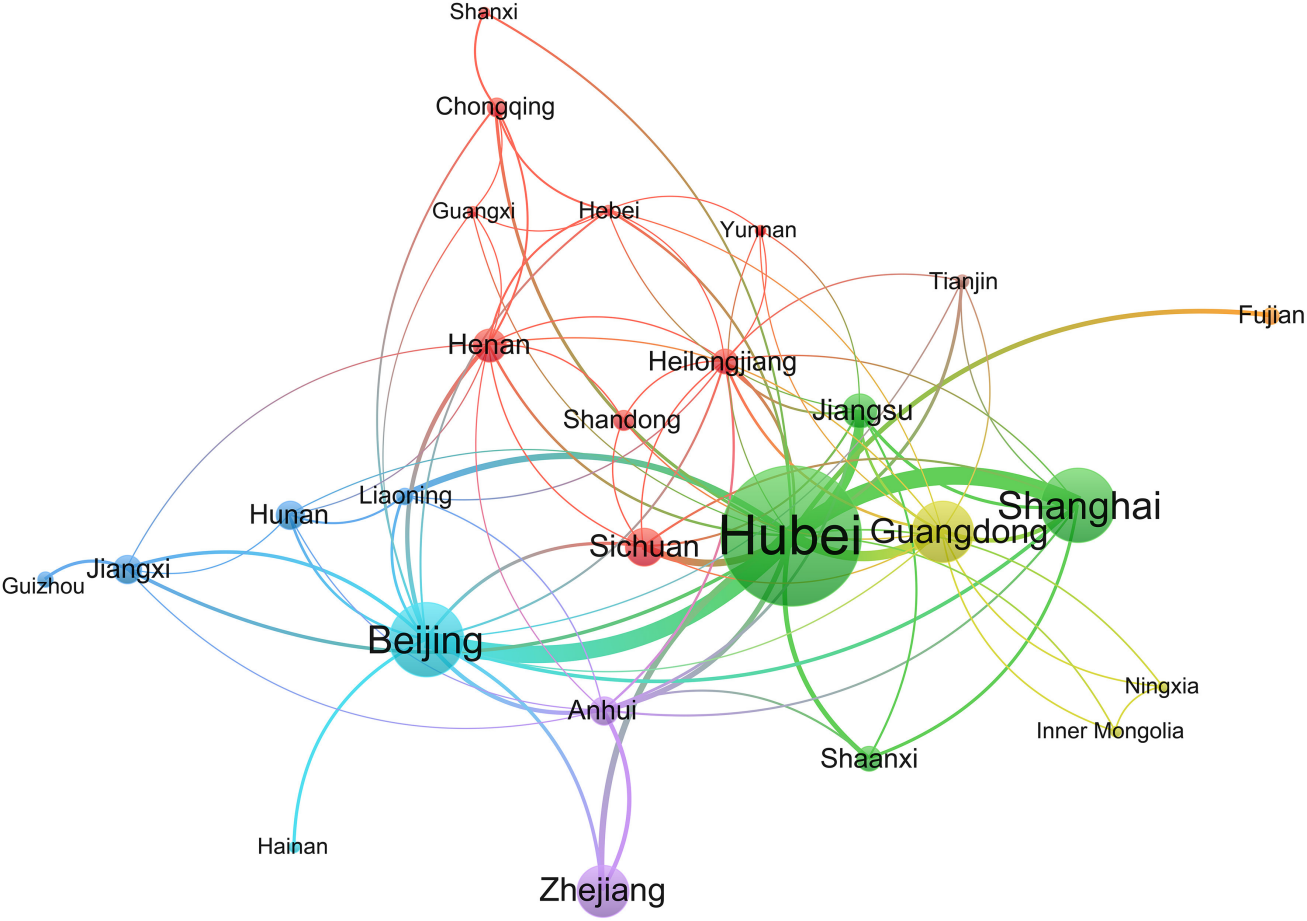
The network map of provinces for registered ICTs of COVID-19.

A social network analysis of provinces revealed that 26 provinces formed a cooperative relationship. Hubei, located in the center of the network, had more collaborations with other provinces. Shanxi, Fujian, Hainan, and Guizhou were situated on the edge of the network and had little cooperation with other provinces. Xinjiang, Jilin, and Hong Kong did not cooperate with other provinces (Figure 2).

### Institutions

A total of 430 institutions contributed to the registration of COVID-19 ICTs. The number of ICTs conducted by one, two, three, four, five, six, seven, eight, nine, and more than nine institutions were 228/390 (58.46%), 78/390 (20.00%), 27/390 (6.92%), 15/390 (3.85%), 14/390 (3.59%), 10/390 (2.56%), 4/390 (1.03%), 4/390 (1.03%), 4/390 (1.03%), and 6/390 (1.54%), respectively. A total of 282/430 (65.58%) institutions participated in only one ICT, and 66/430 (15.35%) institutions participated in two ICTs. Institutions participating in the registration of more than 10 ICTs included Tongji Hospital, Tongji Medical College, Huazhong University of Science and Technology (30/390, 7.69%), Zhongnan Hospital of Wuhan University (18/390, 4.62%), Wuhan Jinyintan Hospital (18/390, 4.62%), Shanghai Public Health Clinical Center (17/390, 4.36%), Union Hospital, Tongji Medical College, Huazhong University of Science and Technology (14/390, 3.59%), the First Affiliated Hospital of Guangzhou Medical University (13/390, 3.33%), Renmin Hospital of Wuhan University (13/390, 3.33%), Guangzhou Eighth People's Hospital (11/390, 2.82%), Huoshenshan Hospital (11/390, 2.82%), and Leishenshan Hospital (11/390, 2.82%), Table 2.

**Table 2:** Institutions contributed to the registration of COVID-19 ICTs (>5) [N (%)].

**Rank**	**Institutions**	**N (%)**
1	Tongji Hospital, Tongji Medical College, Huazhong University of Science and Technology	30 (7.69%)
2	Zhongnan Hospital of Wuhan University	18 (4.62%)
3	Wuhan Jinyintan Hospital	18 (4.62%)
4	Shanghai Public Health Clinical Center	17 (4.36%)
5	Union Hospital, Tongji Medical College, Huazhong University of Science and Technology	14 (3.59%)
6	The First Affiliated Hospital of Guangzhou Medical University	13 (3.33%)
7	Renmin Hospital of Wuhan University	13 (3.33%)
8	Guangzhou Eighth People's Hospital	11 (2.82%)
9	Huoshenshan Hospital	11 (2.82%)
10	Leishenshan Hospital	11 (2.82%)
11	Hubei Integrated Traditional Chinese and Western Medicine Hospital	10 (2.56%)
12	Hubei Provincial Hospital of Traditional Chinese Medicine	10 (2.56%)
13	The First Affiliated Hospital of Zhejiang University School of Medicine	10 (2.56%)
14	Hospital of Chengdu University of Traditional Chinese Medicine	8 (2.05%)
15	Huangshi Hospital of Traditional Chinese Medicine	8 (2.05%)
16	The First Affiliated Hospital of Nanchang University	8 (2.05%)
17	The First Affiliated Hospital of Wenzhou Medical University	8 (2.05%)
18	Beijing You'an Hospital, Capital Medical University	7 (1.79%)
19	West China Hospital of Sichuan University	7 (1.79%)
20	Wuhan Third People's Hospital	7 (1.79%)
21	Wuhan Pulmonary Hospital	7 (1.79%)
22	The First Hospital of Peking University	6 (1.54%)
23	Ruijin Hospital Affiliated to Shanghai Jiaotong University School of Medicine	6 (1.54%)
24	Longhua Hospital Affiliated to Shanghai University of Traditional Chinese Medicine	6 (1.54%)
25	The Third People's Hospital of Shenzhen	6 (1.54%)
26	The Fifth Affiliated Hospital of Sun Yat-Sen University	6 (1.54%)

**Figure 3:**
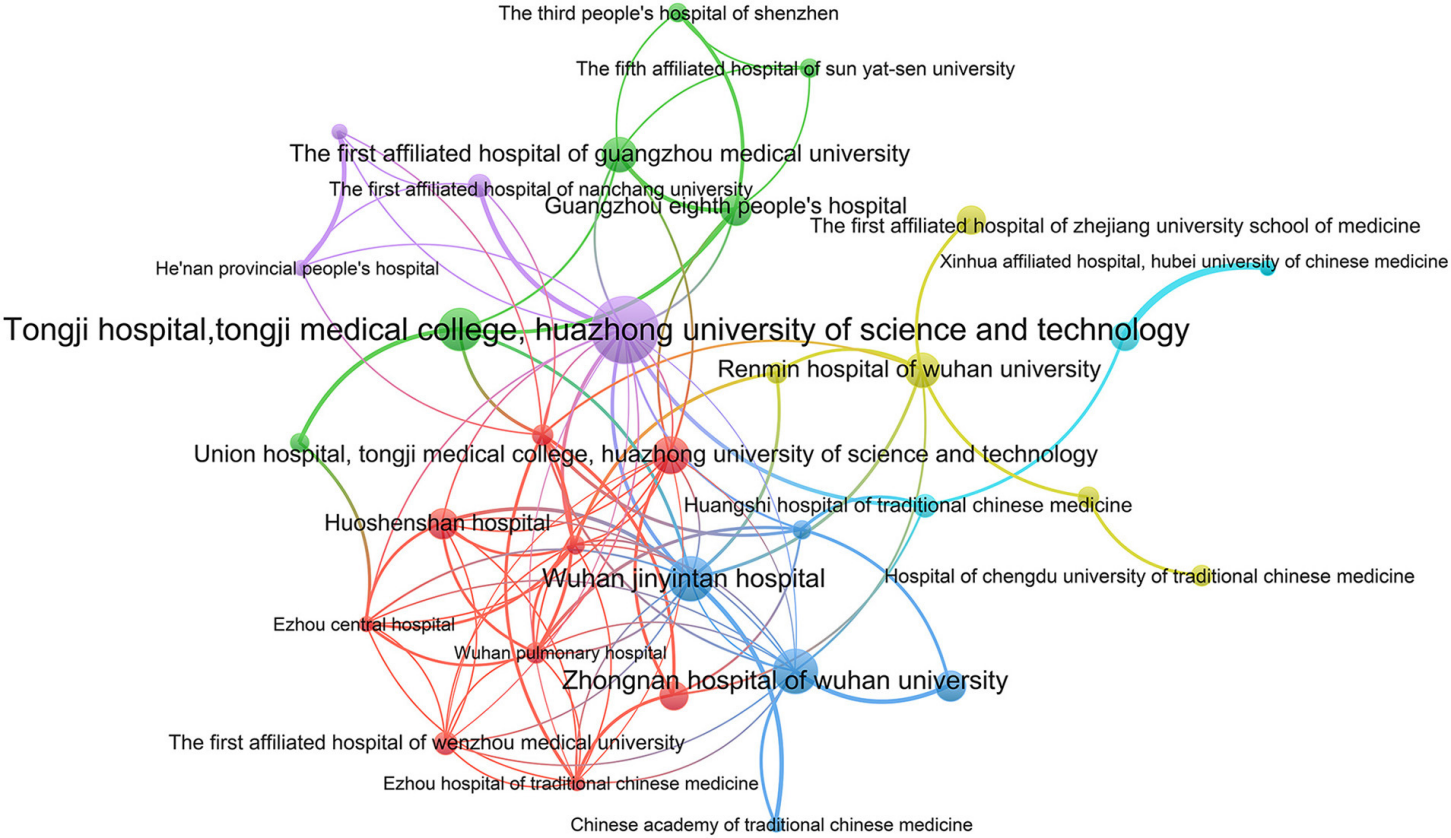
The network map of institutions for registered ICTs of COVID-19.

A cluster analysis was performed for institutions that participated in more than four ICTs. A total of 32 institutions have established cooperative relations and formed six clusters (Figure 3). The largest cooperative team consisted of nine hospitals and research institutions. The smallest team only included three institutions. There was relatively more cooperation between institutions within the team. However, collaboration between different teams was sparse.

### Interventions

There were various types of interventions. Commonly used western medicines included Lopinavir/Ritonavir (34 times), Mesenchymal stem cells (21 times), Interferon α (18 times), Chloroquine phosphate (15 times), Favipiravir (14 times), SARS-COV-2 inactivated/convalescent plasma (10 times), Arbidol (10 times), Thymosin (eight times), Tocilizumab (seven times), Hydroxychloroquine sulfate (six times), and Arbidol hydrochloride (six times). Other western medicines were used less than six times, such as Azvudine, Hydroxychloroquine, Ritonavir, and Remdesivir. A total of 125/390 (32.05%) ICTs focused on traditional Chinese medicine or integrated traditional Chinese and Western medicine, of which 55/390 (14.10%) ICTs mentioned traditional Chinese medicine treatment, traditional Chinese medicine syndrome differentiation treatment, or integrated traditional Chinese and western medicine treatment, but they did not provide specific names of medicine. Among ICTs that provided the specific Chinese medicine, drugs that appeared more than once included Honeysuckle decoction/oral liquid (four times), Xiyanping injection (four times), Shuanghuanglian oral liquid (three times), Lianhua Qingwen capsules/granules (two times), Babaodan (two times), Maxingshigan decoction (two times), Qingfeipaidu decoction (two times), Tanreqing capsule/injection (two times), Xuebijing injection (two times), and Yinhu Qingwen decoction/granules (two times). The remaining Chinese medicines appeared only once, such as Baidu Duan Fang, Bufeihuoxue capsule, Shenqi Fuzheng injection, Fuzheng Huayu tablets, Shenlingbaizhu powder, and Reduning injection.

### Outcome Measures

#### Primary Outcome Measures

**Figure 4:**
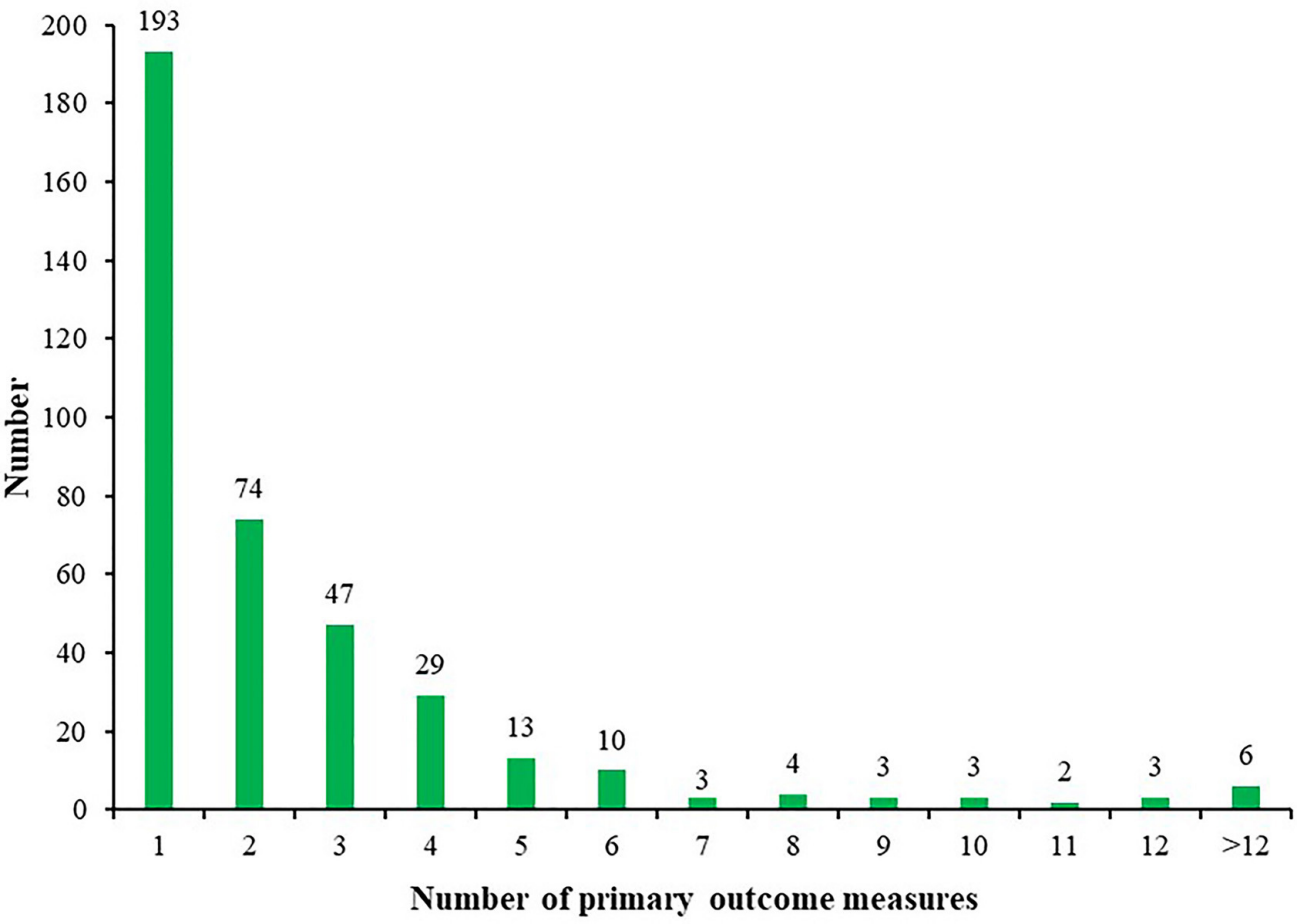
Distribution of the number of primary outcome measures for individual ICT of COVID-19.

**Figure 5:**
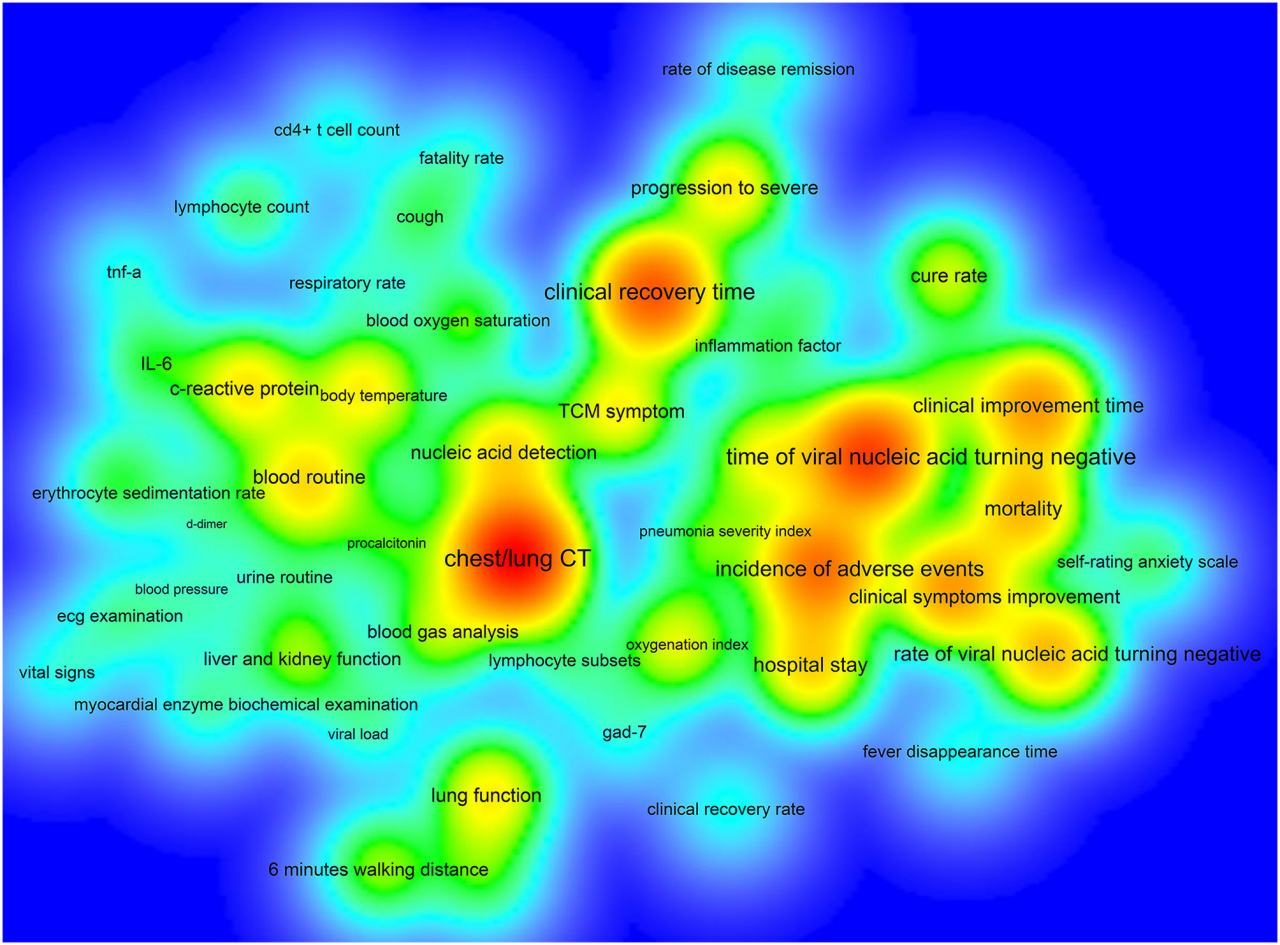
The density map of high-frequency primary outcome measures for registered ICTs of COVID-19.

The number of ICTs with one primary outcome measure was the largest, with 193/390 (49.49%) ICTs. A total of 74/390 (18.97%) ICTs had two primary outcome measures, 47/390 (12.05%) ICTs with three primary outcome measures, and 6/390 (1.54%) ICTs with more than 12 primary outcome measures (Figure 4). Figure 5 shows the primary outcome measures with frequencies greater than two times, which includes 51 outcomes on the map. As shown in Figure 5 and Table 3, chest/lung CT (53/390, 13.59%) was the most commonly used primary outcome measure, followed by the time of viral nucleic acid turning negative (40/390, 10.26%), clinical recovery time (35/390, 8.97%), incidence of adverse events (30/390, 7.69%), clinical improvement time (23/390, 5.90%), clinical symptoms improvement (23/390, 5.90%), mortality (19/390, 4.87%), rate of viral nucleic acid turning negative (19/390, 4.87%), hospital stay (16/390, 4.10%), and blood routine (15/390, 3.85%).

**Table 3:** The top 20 primary outcome measures in terms of frequency [N (%)].

**Rank**	**Primary outcome measures**	**N (%)**	**Rank**	**Primary outcome measures**	**N (%)**
1	Chest/lung CT	53 (13.59%)	11	Nucleic acid detection	15 (3.85%)
2	Time of viral nucleic acid turning negative	40 (10.26%)	12	C-reactive protein	14 (3.59%)
3	Clinical recovery time	35 (8.97%)	13	Rate of progression to severe	14 (3.59%)
4	Incidence of adverse events	30 (7.69%)	14	Body temperature	13 (3.33%)
5	Clinical improvement time	23 (5.90%)	15	Lung function	13 (3.33%)
6	Clinical symptoms improvement	23 (5.90%)	16	TCM symptom	13 (3.33%)
7	Mortality	19 (4.87%)	17	Antipyretic time	12 (3.08%)
8	Rate of viral nucleic acid turning negative	19 (4.87%)	18	Oxygenation index	11 (2.82%)
9	Hospital stay	16 (4.10%)	19	Cure rate	10 (2.56%)
10	Blood routine	15 (3.85%)	20	Blood gas analysis	9 (2.31%)

#### Secondary Outcome Measures

**Figure 6:**
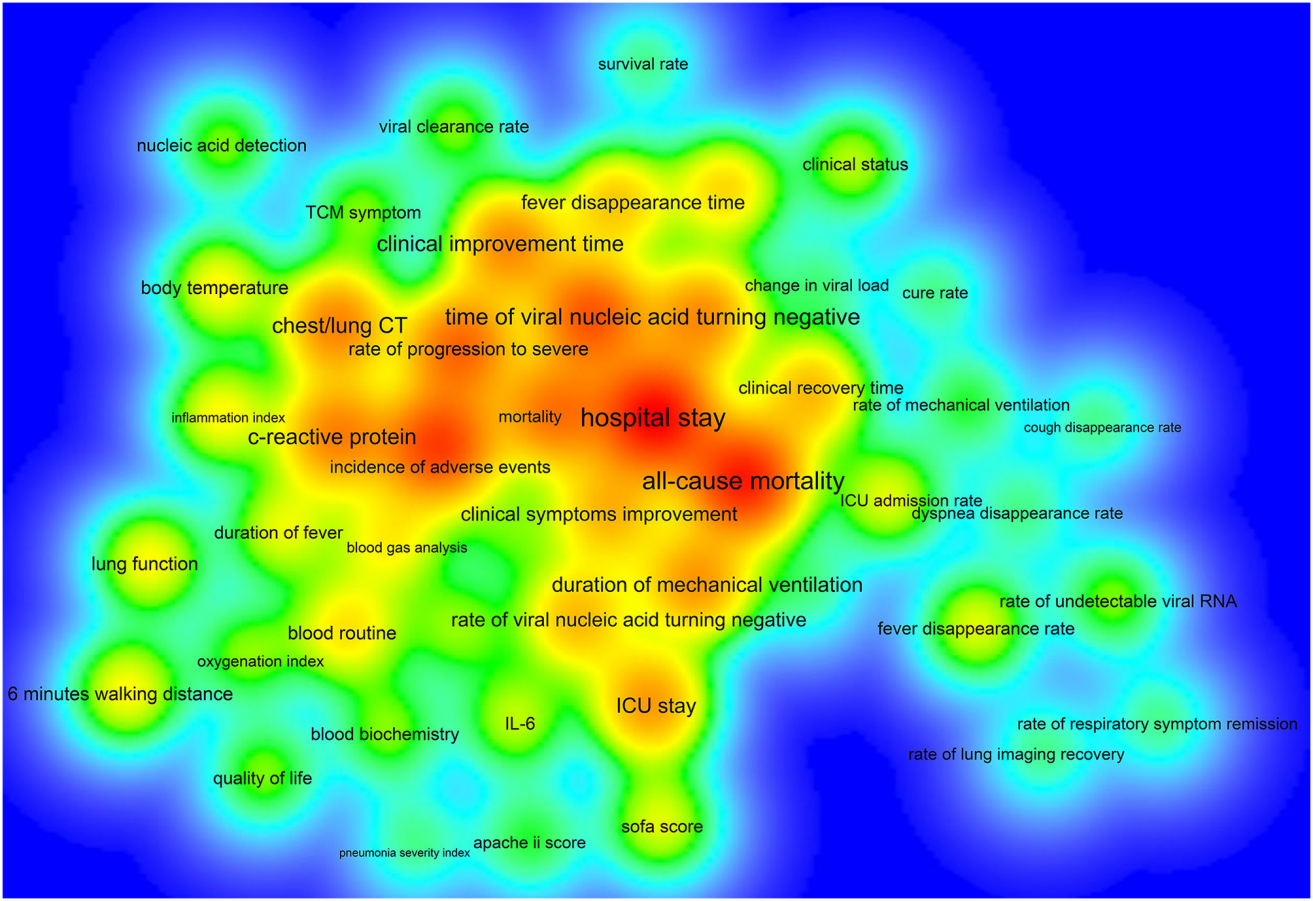
The density map of high-frequency secondary outcome measures for registered ICTs of COVID-19.

Of the 390 ICTs, 279 (71.54%) ICTs have secondary outcomes. Figure 6 shows the secondary outcome measures with frequencies greater than two times, which includes 49 outcomes on the map. Hospital stay (33/390, 8.46%) was the most commonly used secondary outcome measure, followed by all-cause mortality (30/390, 7.69%), incidence of adverse events (25/390, 6.41%), time of viral nucleic acid turning negative (22/390, 5.64%), rate of progression to severe (20/390, 5.13%), mortality (18/390, 4.62%), chest/lung CT (17/390, 4.36%), C-reactive protein (17/390, 4.36%), clinical improvement time (16/390, 4.10%), and incidence of serious adverse events (16/390, 4.10%), Table 4.

**Table 4:** The top 20 secondary outcome measures in terms of frequency [N (%)].

**Rank**	**Secondary outcome measures**	**N (%)**	**Rank**	**Secondary outcome measures**	**N (%)**
1	Hospital stay	33 (8.46%)	11	Duration of mechanical ventilation	15 (3.85%)
2	All-cause mortality	30 (7.69%)	12	ICU stay	15 (3.85%)
3	Incidence of adverse events	25 (6.41%)	13	Clinical recovery time	12 (3.08%)
4	Time of viral nucleic acid turning negative	22 (5.64%)	14	Clinical symptoms improvement	12 (3.08%)
5	Rate of progression to severe	20 (5.13%)	15	Rate of viral nucleic acid turning negative	12 (3.08%)
6	Mortality	18 (4.62%)	16	Fever disappearance time	11 (2.82%)
7	Chest/lung CT	17 (4.36%)	17	Duration of supplemental oxygenation	10 (2.56%)
8	C-reactive protein	17 (4.36%)	18	Blood routine	9 (2.31%)
9	Clinical improvement time	16 (4.10%)	19	Blood gas analysis	8 (2.05%)
10	Incidence of serious adverse events	16 (4.10%)	20	Body temperature	8 (2.05%)

## Discussion

A total of 29 provinces from China contributed to the registration of COVID-19 ICTs, of which 55.17% provinces participated in <10 ICTs, while Hubei province participated in 160 ICTs, indicating that ICTs registrations were mainly concentrated in a few provinces. Through the network analysis of provinces, we found that Hubei and Beijing had more collaborations with other provinces, but the collaborations between the remaining provinces were not close. A total of 430 institutions participated in the registration of COVID-19 ICTs, but only 26 institutions participated in the registration of more than five ICTs, and 80.93% of the institutions contributed to only one or two ICTs. The productive institutions formed six cooperative teams and the number of institutions within the teams did not exceed nine. The cooperation between institutions within each team was relatively close, but cooperation between different teams was sparse. Therefore, future researchers should strengthen more comprehensive and extensive cooperation between different provinces and different regions. Through the analysis of the sample size, we found that the sample size of 26.67% ICTs was lower than 50. Some ICTs only included 10 patients, which were inadequate. 12.82% of ICTs had a sample size > 300, with the maximum sample size up to 20,000, but the sample size of some ICTs was too large to be performed in just one institution, as the sample size far exceeds the total number of patients in their region. However, they did not carry out cross-institutional and cross-regional cooperation. Besides, patients before the trial should be ruled out, which shows that it is difficult to complete the trial according to the research protocol. This also shows that it is necessary to strengthen cooperation and exchanges and carry out multi-center research.

In clinical trials, many strategies have been tried to treat COVID-19. Although there is no specific drug for COVID-19 (15), the drug used in clinical trials should also be carefully chosen to avoid additional damage to the patient's health. The commonly studied western medicines included Lopinavir/Ritonavir, Mesenchymal stem cells, Interferon α, Chloroquine phosphate, Hydroxychloroquine sulfate, Favipiravir, and Arbidol. However, the sample sizes of many trials were insufficient, and the usage, dosage, and treatment course of drugs were unclear, which may lead to a lack of credibility in the results of the research. Therefore, future researchers should conduct large-scale, multi-center clinical trials, rather than repeating trials for an intervention, to avoid wasting resources. Of the 125 ICTs concerned with traditional Chinese medicine or integrated traditional Chinese and Western medicine, about 45.00% of the trials did not provide specific names and usages of traditional Chinese medicine. Besides, the most commonly used control was the usual treatment, but most ICTs did not provide specific content of the usual treatment. Future trial registers and reviewers of registry platforms should pay more attention to these aspects to promote the registration of COVID-19 clinical trials more standardized.

Some ICTs only adopted one primary outcome measure, and some ICTs had more than 12 primary outcome measures, which indicated that there was a considerable difference in the number of primary outcomes. Chest/lung CT, time of viral nucleic acid turning negative, the incidence of adverse events, clinical improvement time, mortality, and hospital stay were among the top 10 primary outcomes, as well as among the top ten secondary outcomes, indicating that these six outcome measures were key outcomes in this field. Future researchers can use these measures when conducting COVID-19 clinical trials. This study found that there are some problems with the outcome measures: (1) there were too many types of indicators and lack of main outcome measures, which added difficulties to the development of systematic reviews and guidelines; (2) the expression of outcome measures was not standardized, and there were multiple expression terms for the same measure; (3) the definitions of outcome measures were not clear, and many outcome measures were ambiguous; (4) most ICTs did not clarify the time of follow-up and the measurement time of the outcomes; (5) the selected outcome measures cannot fully reflect the expected research results; (6) regarding outcomes that need to be measured, most ICTs did not provide measurement tools; and (7), considering ICTs that focused on the traditional Chinese medicine and integrated traditional Chinese and Western medicine, there was a lack of outcome measures with characteristics of traditional Chinese medicine. These shortcomings need to be further improved for future clinical trials of COVID-19.

We conducted a comprehensive analysis of the registered ICTs of COVID-19 conducted in China using the bibliometric analysis method and presented collaborations of provinces and institutions, and the distribution of outcome measures by using visual network maps and density maps. However, this study also has some limitations. Firstly, only ICTs from China were included, and many clinical trials will be registered in the future, which cannot fully reflect the status of all clinical trials and may not apply to ICTs in other countries. Secondly, since some institutions, interventions, and outcomes have different expressions, although we have standardized them, bias may still exist. Thirdly, some registered ICTs may not provide all participating institutions, resulting in the results of this study may differ from the actual situation. Finally, since this study was based on data of registered ICTs, we did not explore the effectiveness of the interventions and outcome measures. Further studies are needed to assess whether the registered ICTs have been completed and whether the interventions and outcome measures studied are effective.

During the COVID-19 pandemic, we are very pleased that scholars from all over the world are actively conducting clinical trials to explore effective drugs for the treatment of COVID-19. However, our study found that the registered ICTs had many defects in methods and results. Therefore, future researchers should optimize the methods of these trials and ensure the transparency of their methods to produce high-quality evidence. Otherwise, it will not only result in a waste of resources and property, but more importantly, mislead the measures to deal with COVID-19 and delay treatment for patients. Furthermore, researchers should facilitate the completion of these clinical trials and translate the results of these trials into practices and policies.

## Conclusions

The number of daily registrations for ICTs of COVID-19 fluctuated significantly. Hubei, Shanghai, and Beijing are the top three productive provinces. Tongji Hospital, Tongji Medical College, Huazhong University of Science and Technology, Zhongnan Hospital of Wuhan University, and Wuhan Jinyintan Hospital are the top three productive institutions. Collaborations between provinces and institutions were not close enough. More comprehensive and extensive collaborations between different provinces and different regions should be further strengthened. The identified deficiencies in interventions and outcome measures should be given more attention by future researchers of COVID-19.

## Author Contributions

YG and JT planned and designed the study. YG, KY, ML, YC, and SS participated in the literature search and data collection. YG, KY, ML, and FY analyzed the data. YG and JT drafted the manuscript. YG, FY, and JT revised the manuscript. All authors read and approved the final manuscript.

## Conflict of Interest

The authors declare that the research was conducted in the absence of any commercial or financial relationships that could be construed as a potential conflict of interest.

## Supplementary Material

Figure S1 The flowchart of the screening process.

## Abbreviations

COVID-19: Corona Virus Disease 2019
SARS-CoV-2: Severe acute respiratory syndrome coronavirus 2
ICTs: interventional clinical trials.
