# Supplementary figures and images for: Research Collaboration and Outcome Measures of Interventional Clinical Trial Protocols for COVID-19 in China
Source: Front Public Health. 2020 Sep 2;8:554247. doi: 10.3389/fpubh.2020.554247 (PMC7492615; doi:10.3389/fpubh.2020.554247)

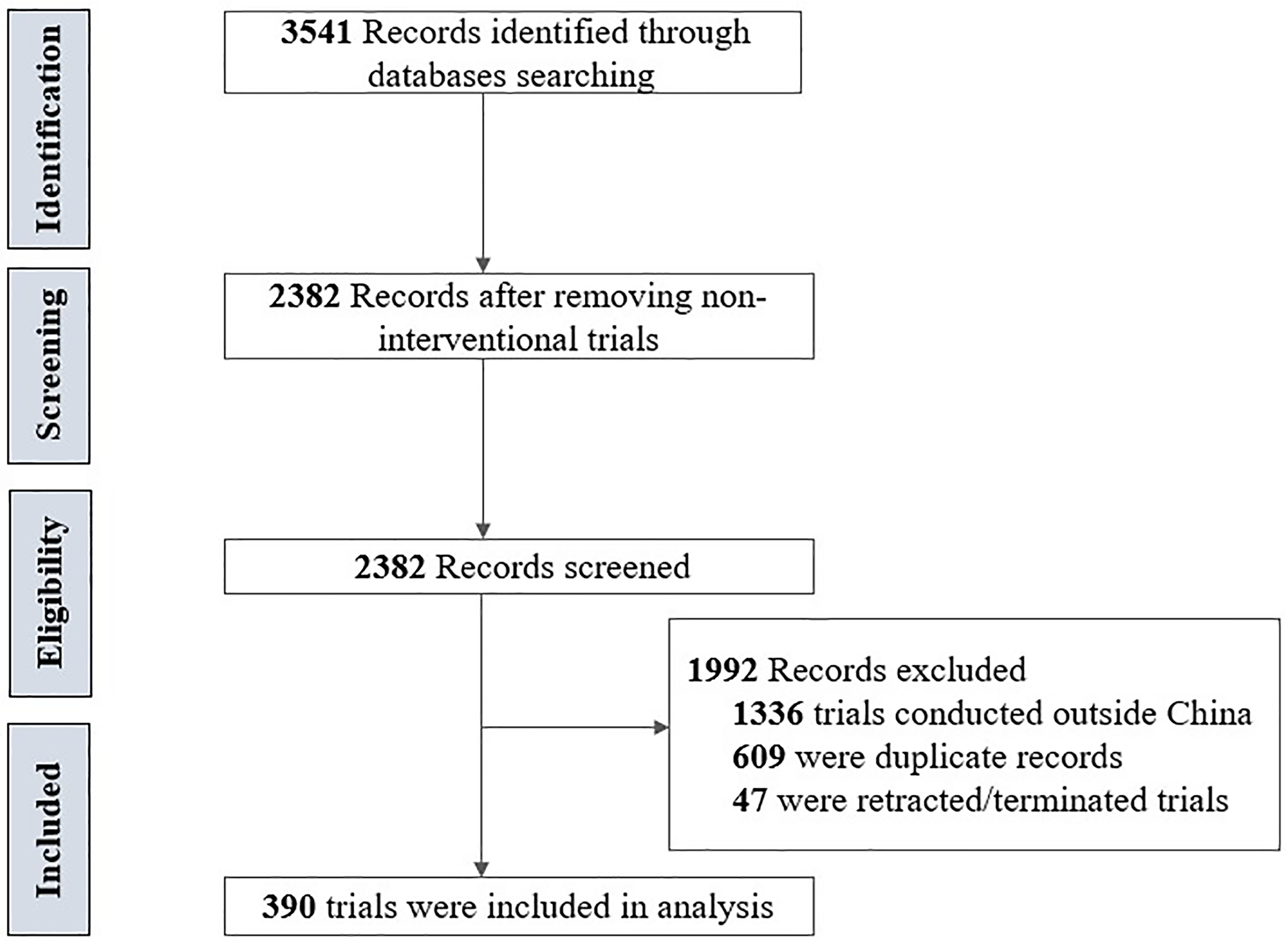

Supplement: Figure S1 — The flowchart of the screening process. [file Image_1.TIF]
